# Synthesized Magnolol Derivatives Improve Anti-*Micropterus salmoides* Rhabdovirus (MSRV) Activity In Vivo

**DOI:** 10.3390/v14071421

**Published:** 2022-06-28

**Authors:** Yingjie Jin, Fei Yang, Gengrong Zhang, Qing Yu, Gaoxue Wang, Fei Ling, Tianqiang Liu

**Affiliations:** 1 College of Animal Science and Technology, Northwest A&F University, Xinong Road 22nd, Yangling 712100, China; jinyingjie0015@163.com (Y.J.); fhy1849@163.com (F.Y.); wanggaoxue@126.com (G.W.); 2 Shenzhen Research Institute, Northwest A&F University, Gaoxin South 4th Road, Shenzhen Virtual University Park Building, High-Tech Industrial Park, Shenzhen 518057, China; zhanggengrong@nwafu.edu.cn; 3 Guangxi Engineering Research Center for Fishery Major Diseases Control and Efficient Healthy Breeding Industrial Technology (GERCFT), Guangxi Key Laboratory of Aquatic Biotechnology and Modern Ecological Aquaculture, Guangxi Academy of Sciences, Nanning 530007, China; yu_qing1990@163.com

**Keywords:** *Micropterus salmoides* rhabdovirus (MSRV), magnolol, largemouth bass, antiviral activity

## Abstract

*Micropterus salmoides* rhabdovirus (MSRV) is a primary viral pathogen in largemouth bass aquaculture, which leads to tremendous economic losses yearly. Currently, there are no approved drugs for the treatment and control of this virus. Our previous studies screened the herb *Magnolia officinalis* from many traditional Chinese medicines, and we isolated and identified magnolol as its main active compound against multiple rhabdoviruses, including MSRV. On the basis of the structure–activity relationship and pharmacophore model of magnolol, two new magnolol derivatives, namely, hydrogenated magnolol and 2,2′-dimethoxy-magnolol, were designed and synthesized. Their anti-MSRV activities were systematically investigated both in vitro and in vivo. By comparing the half-maximal inhibitory concentration (IC_50_), it was found that hydrogenated magnolol possessed a higher anti-MSRV activity than magnolol and 2,2′-dimethoxy-magnolol, with an IC_50_ of 13.37 μM. Furthermore, hydrogenated magnolol exhibited a protective effect on the grass carp ovary (GCO) cell line by reducing the cytopathic effect induced by MSRV. Further studies revealed that hydrogenated magnolol did not directly impact virions or interfere with MSRV adsorption. It worked within the 6–8 h of the phase of virus replication. In vivo treatment of MSRV infection with magnolol and hydrogenated magnolol showed that they significantly improved the survival rate by 44.6% and 62.7%, respectively, compared to MSRV-infected groups. The viral load measured by the expression of viral glycoprotein in the organs including the liver, spleen, and kidney also significantly decreased when fish were intraperitoneally injected at a dose of 20 mg/kg. Altogether, the structural optimization of magnolol via hydrogenation of the propylene groups increased its anti-MSRV activity both in vitro and in vivo. These results may provide a valuable reference for anti-MSRV drug discovery and development in aquaculture.

## 1. Introduction

Largemouth bass (*Micropterus salmoides*) have been cultured in China since being introduced into the Guangdong province of China from the United States in 1983, and they are considered one of the most popular warm-water fish in China [1]. Due to their popularity as a sport fish and food fish, they have been stocked across North, Central, and South America, Europe, and Asia [2]. In 2020, the production of largemouth bass was 61,9519 tons, being the eighth largest farmed fish species in China [3]. However, with the fast development of intensive fish farming and high stocking densities, largemouth bass farming has suffered from epidemic infectious diseases caused by organisms such as bacteria, fungi, viruses, or parasites. Among these pathogens, a lethal viral disease caused by *Micropterus salmoides* rhabdovirus (MSRV) results in tremendous economic losses to the industry. Outbreaks of MSRV could cause symptoms such as spiral, erratic swimming, distorted bodies, and bloated abdomen [4]. What is most important is that MSRV infection can lead to high mortality of fish. In April 2011, MSRV infection caused about 200,000 deaths of largemouth bass fingerlings (2.5–4.5 cm in body length) in a fish farm located in Zhongshan city, Guangdong Province, China [5]. However, no commercially approved drug is available for treating this viral infection. Thus, the development of effective methods for the prevention and control of MSRV disease is in great demand. 

Remarkable progress has been achieved in the field of herbal therapy owing to the increasing concerns of drug resistance development and limited advances in the field of antiviral drug discovery. In many civilizations, natural compounds from herbs have been used for the treatment of viral diseases for centuries, and they play a vital role in modern medicinal development because of their broad therapeutic spectrum and minimal or no side-effects [6]. As synthetic antiviral drugs are not available against most viral pathogens, all possible efforts have been focused on the search for novel pharmaceuticals to treat viral infection through the isolation, characterization, and synthesis of pharmacologically actives from natural plants. In aquaculture, arctigenin (from *Arctium lappa* L.), bavachin (from *Psoralea coryfolia*), and saikosaponin D (*Bupleurum kunmingense*) showed high antiviral efficacy against spring viremia of carp virus (SVCV) in epithelioma papulosum cyprini (EPC) cells [7,8,9]. Ursolic acid extracted from *Prunella vulgaris* L. inhibited infectious hematopoietic necrosis virus (IHNV) replication with a maximum inhibitory percentage >90% [10]. More importantly, magnolol, a natural product identified from *Magnolia officinalis* Rehd et Wils., was found by our laboratory, which shows efficient antiviral bioactivity against grass carp reovirus (GCRV), by suppressing viral replication and protecting cells from apoptosis [11,12]. Furthermore, magnolol also showed admirable anti-*Saprolegnia* activity at a concentration of 9.2 mg/L [13]. In the abovementioned published research, we synthesized six magnolol derivatives and investigated their structure–activity relationship (SAR) by testing their anti-*Saprolegnia* activity. Together with the pharmacophore model using the Phase module of Schrodinger software (Schrodinger LLC, New York, NY, USA) [14], it was revealed that the phenolic hydroxyl groups and propylene groups of magnolol contribute greatly to the hydrogen bond acceptor and hydrophobic interactions, respectively (Figure S1). Thus, in the present study, we synthesized two magnolol derivatives on the basis of our previous studies to conduct further structural optimization and biological evaluation assays against MSRV.

**Figure 1:**
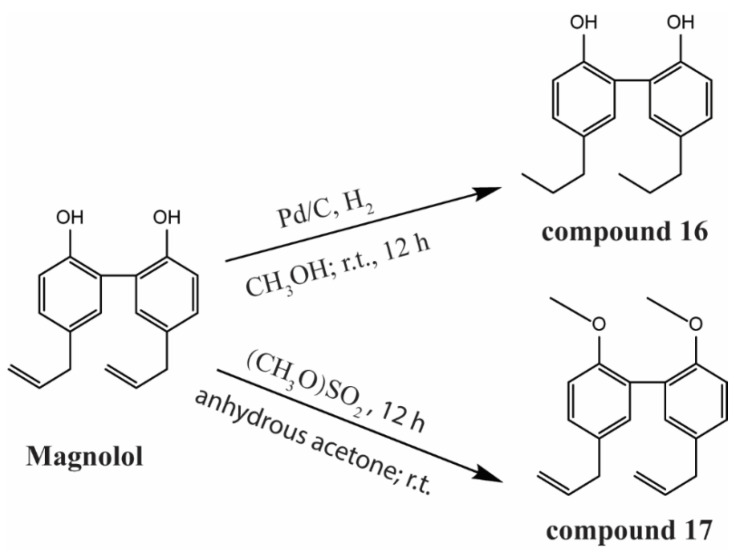
Reaction scheme showing the synthesis of compound **16** and compound **17** from magnolol.

The chemical structures and general reaction scheme of magnolol and two magnolol derivatives, namely, 5,5′-dipropyl-[1,1′-biphenyl]-2,2′-diol (compound **16**) and 2,2′-dimethoxy-5,5′-diallyl-biphenyl (compound **17**), are shown in Figure 1. Their anti-MSRV activities were analyzed by real-time quantitative PCR (qRT-PCR). Moreover, virus titration assay, cytopathic effect (CPE) reduction analysis, and nucleus damage observation were applied to validate their antiviral activity. In addition, the mechanism of action of magnolol and its derivatives were investigated by studying the effect of magnolol on each stage of MSRV virion proliferation. Subsequently, the anti-MSRV activity of magnolol was evaluated in vivo as a function of the viral load and survival of largemouth bass fingerlings. The present study further explores the structural optimization of magnolol for the treatment of MSRV, and sets the foundation for antiviral drug development in aquaculture. 

## 2. Materials and Methods

### 2.1. MSRV Virus Strain, Cell Lines, and Largemouth Bass

The MSRV strain (MSRV-FJ985) was isolated, identified, and stored in our laboratory with GenBank access no. MT818233.1 [15]. The grass carp ovary (GCO) cells were kindly provided by the Zhejiang Institute of Freshwater Fisheries (Huzhou, China). Cells were cultured at 25 °C under humidified air/5% CO_2_ in medium 199 (Hyclone, Grand Island, NY, USA) containing 10% fetal bovine serum (FBS, Zeta Life, Menlo Park, CA, USA), streptomycin (100 U/mL), and penicillin (100 U/mL). Largemouth bass fingerlings (*n* = 1000, 4.72 ± 0.46 cm in length, 1.34 ± 0.35 g in weight) were bought from an Aquatic Animal Breeding Farm in Chongqing, China and confirmed to be MSRV-free for the experiments by qRT-PCR. Largemouth bass were maintained in a recirculating system at 25 °C, pH of 6.8–8.0, and dissolved oxygen >5.7 mg/L. Fish were fed with commercial fodder (Fuxing Organism Co., Ltd., Fuzhou, China) twice a day and accommodated for 3 weeks prior to experiments. 

### 2.2. Reagents

Magnolol (>99% purity) was purchased from Aladdin Biochemical Technology (Shanghai, China). Other chemicals were obtained from Sigma-Aldrich (St. Louis, MO, USA) and used without further purification. Anhydrous acetone, methanol, and dimethyl sulfate (DMS) were bought from Sinopharm Chemical Reagent Co., Ltd. (Beijing, China). Silica gel H (200–300 mesh, Qingdao Marine Chemical Factory, Qingdao, China) was applied for column chromatography. Silica gel (cat no. GF254, Qingdao Marine Chemical Factory, Qingdao, China) was used for thin-layer chromatography (TLC). Milli-Q water (Millipore, Burlington, MA, USA) was used to make aqueous solutions. 

### 2.3. Synthesis of Magnolol Derivatives ***16*** and ***17***

As shown in Figure 1, magnolol solution (1.00 g, 3.75 mmol) in 30 mL of methanol with 10% Pd/C (100 mg, ca. 0.1 mmol of Pd metal) was stirred under atmospheric H_2_ pressure at room temperature. After reacting for 12 h, the product (compound **16**) was dried and collected for nuclear magnetic resonance (NMR) characterization. For the synthesis of compound **17**, 1.07 g magnolol (4.07 mmol) was added to 30 mL of anhydrous acetone and 2.76 g of anhydrous K_2_CO_3_ (19.97 mmol) at room temperature (r.t.). The mixture was stirred vigorously for 30 min, followed by adding 0.77 mL of DMS, before reacting for another 12 h. Crude products were washed with 30 mL of acetone and extracted with ethyl acetate three times. The organic layer was combined, dried with anhydrous Na_2_SO_4_, and purified via silica gel column chromatography with mixed petroleum ether and ethyl acetate (8:1, *v*/*v*) as eluent. The elution was collected and evaporated under reduced pressure to obtain compound **17**. 

### 2.4. Cytotoxicity of Magnolol and Compounds ***16*** and ***17*** toward GCO Cells

For the cytotoxicity assay of magnolol derivatives **16** and **17**, GCO cells were grown to a 90% confluent layer in 96-well plates. Then, cells were treated with different concentrations of magnolol (7.51, 15.02, 18.77, 30.04, and 38.86 μM), compound **16** (5.92, 11.10, 14.79, 22.19, and 28.89 μM), and compound **17** (135.87, 271.75, 543.50, 679.37, 815.25, and 1019.06 μM) for 48 h. The cell viability assay was conducted using a CCK-8 kit (Beyotime Biotechnology, China) according to the manufacturer’s introduction. A 20% cytotoxic concentration (CC_20_) was regarded as the highest safe concentration according to other researchers’ studies [16].

### 2.5. Anti-MSRV Activity of Magnolol and Magnolol Derivatives ***16*** and ***17*** In Vitro

To measure the anti-MSRV activity of **16** and **17**, GCO cells were seeded into a 12-well plate and cultured overnight. After changing the cell culture medium, freshly prepared cell culture medium containing 1 × 10^3^ TCID_50_ MSRV was added and incubated for 2 h. Then, the cell culture medium was pipetted out and washed with PBS (pH 7.2) three times. Subsequently, GCO cells were maintained for 2 days with a cell culture medium containing magnolol, compound **16**, or compound **17** at the concentration of CC_20_. The expression of MSRV glycoprotein (G protein) was detected to calculate viral load by qRT-PCR. 

### 2.6. RNA Isolation, cDNA Transcription, and qRT-PCR Assay

Total RNA was extracted from harvested GCO cells using Trizol (Accurate Biology Co. Ltd., Shenzhen, China) according to the manufacturer’s protocol. The quality and the purity of isolated RNA were determined using a Nanodrop 2000c spectrophotometer (NanoDrop Technologies, Wilmington, DE, USA). RNA was reverse-transcribed to cDNA using the HiScript Q Select RT Supermix kit (Vazyme, Nanjing, China) for qPCR (+gDNA wiper). The qRT-PCR protocol was as follows: 95 °C for 4 min, and then 38 cycles at 95 °C denaturation for 10 s, followed by annealing at 59 °C for 30 s with a Bio-Rad iCycler IQ5 Multicolor real time PCR detection system. Table 1 shows the primers used for qRT-PCR in the study. 

### 2.7. Anti-MSRV Activity of Magnolol and Magnolol Derivative ***16*** In Vitro

To investigate the antiviral activity of magnolol and compound **16** on the basis of the antiviral activity screening in Section 2.5, a dose–effect assay of magnolol and compound **16** against MSRV in GCO cells was carried out. GCO cells at 90% confluence were infected with MSRV (1 × 10^3^ TCID_50_) and treated with magnolol or compound **16** at six different concentrations, ranging from 5.92 μM to 38.86 μM. The viral load of GCO cells was measured following the methods described in Section 2.5. Furthermore, the CPE and the number of cytopathic cells were tracked and recorded every day for 4 days. 

### 2.8. Fluorescence Microscopy Observation of Cell Nucleus Damage 

Inoculated GCO cells were seeded on glass coverslips in a 12-well cell culture plate, and cultured overnight to form a monolayer. Next, the cells were incubated with MSRV and compounds for 48 h. Then, the cells were washed with PBS buffer three times and fixed with 4% paraformaldehyde fixing solution for 20 min. Cell nuclei were stained with DAPI (Beyotime, Shanghai, China) for 20 min according to the manufacturer’s instructions and imaged using an upright fluorescence microscope (Leica DM5000, Wetzlar, Germany).

### 2.9. Effect of Hydrogenated Magnolol on MSRV Infection Steps in Host Cells

To study the effect of hydrogenated magnolol on MSRV infection steps in GCO cells, four different experimental setups were designed. The first assay was scheduled to evaluate whether hydrogenated magnolol could directly interact with the virions. MSRV (1 × 10^3^ TCID_50_) and hydrogenated magnolol (28.89 μM) were coincubated for 1, 2, and 4 h at 25 °C. Then, the viral particles were collected by ultracentrifugation (33,000 rpm, 4 °C, 2 h) using an overspeed refrigerated centrifuge (Optima XPN-100, Beckman, Palo Alto, CA, USA). Then, GCO cells were infected with the collected virus and incubated for 48 h before determining the viral load by qRT-PCR. In addition, the effect of hydrogenated magnolol on virus adsorption by GCO cells was investigated. Specifically, 90% confluent GCO cells in a 25 cm^2^ cell culture flask were infected with MSRV (1 × 10^3^ TCID_50_) with or without hydrogenated magnolol (28.89 μM) for 30 min at 4 °C to allow MSRV binding but not internalization and replication. Then the viral load was measured using the method described above. Furthermore, a time-of-addition study was performed to analyze the effect of hydrogenated magnolol on different phases of the virus replication cycle. GCO cells in a monolayer were infected with MSRV (1 × 10^3^ TCID_50_) for 2 h and exposed to hydrogenated magnolol (28.89 μM) after 1, 2, and 4 h. After being coincubated for another 2 h, hydrogenated magnolol was removed, and cells were washed with PBS three times prior to 24 h culture with maintenance medium. The same method was applied to determine the viral load. A fourth assay was designed to evaluate whether hydrogenated magnolol could inhibit the release of MSRV virions from host cells. GCO cells were seeded into a 12-well plate to a monolayer. After being infected with MSRV (1 × 10^3^ TCID_50_) for 2 h, the medium was replaced with maintenance medium containing hydrogenated magnolol (28.89 μM), and cell supernatants were collected to determine the viral titer after incubating for 48 h. Viral titer was calculated using the Spearman–Karber method [17].

### 2.10. In Vivo Anti-MSRV Activity of Hydrogenated Magnolol 

A total of 120 healthy largemouth bass were randomly divided into four groups (30 fish/group), named groups A, B, C, and D. Fish in groups A, B, C, and D were intraperitoneally injected with 20 μL of PBS (control group), MSRV (1 × 10^3^ TCID_50_), MSRV (1 × 10^3^ TCID_50_) and magnolol (20 mg/kg), or MSRV (1 × 10^3^ TCID_50_) and hydrogenated magnolol (20 mg/kg), respectively. The survival rate of fish in each group was recorded daily for 14 days post injection. Three fish were randomly sampled on days 1, 3, 5, and 7 post injection. The liver, spleen, and kidney of each fish were collected for viral load determination by analyzing the expression of MSRV G protein. 

### 2.11. Statistical Analysis

The half-maximal inhibitory concentration (IC_50_) of the magnolol derivatives was calculated using the Probit regression model (SPSS, IBM company, USA). Data were analyzed using an unpaired, two-tailed Student’s *t*-test or one-way ANOVA after normalization to determine significance. All data are presented as the mean ± SEM (standard error of the mean). The differences were determined by LSD test and were considered as significant at * *p* < 0.05 and very significant at ** *p* < 0.01.

## 3. Results 

### 3.1. Synthetization and Characterization of Magnolol Derivatives

**Figure 2:**
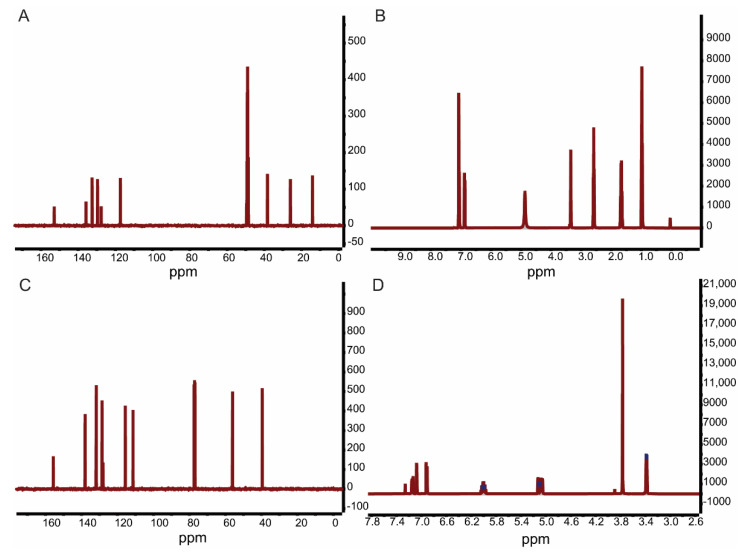
^13^C– and ^1^H–NMR spectra of hydrogenated magnolol (**A**,**B**) and 2,2′–dimethoxy-magnolol (**C**,**D**).

As shown in Figure 2, the ^13^C- and ^1^H-NMR spectra of hydrogenated magnolol yielded the following results: ^13^C-NMR (126 MHz, Me OD): δ 152.88, 135.76, 132.52, 129.57, 127.64, 117.32, 38.30, 25.99, 14.09; ^1^H-NMR (CDCl_3_, 500 MHz): δ 7.06 (d, J = 9.5 Hz, 4H), 6.88(d, J = 7.9 Hz, 2H), 2.58 (t, J = 7.3 Hz, 4H), 1.68 (dt, J = 14.5,7.1 Hz, 4H), 0.98 (t, J = 7.1 Hz, 6H). The ^13^C- and ^1^H-NMR spectra of 2,2′-dimethoxy-magnolol yielded the following results: ^13^C-NMR (126 MHz, CDCl3): δ 155.65, 137.98, 131.91, 131.74, 128.56, 128.01, 115.62, 111.32, 56.03, 39.54; ^1^H-NMR (CDCl_3_, 500 MHz): δ 7.14 (dd, J = 8.4, 2.2 Hz, 2H),7.07 (d, J = 2.1 Hz, 2H), 6.91 (d, J = 8.4 Hz, 2H), 6.00 (ddt, J = 16.8, 10.0, 6.8 Hz, 2H), 5.04–5.14 (m, 4H), 3.77 (s, 6H), 3.38 (d, J = 6.7 Hz, 4H). According to a comparison with NMR data from other studies, the magnolol derivatives were successfully synthesized. 

### 3.2. Cytotoxicity and Anti-MSRV Activities of Magnolol and Compounds ***16*** and ***17*** on GCO Cells

**Figure 3:**
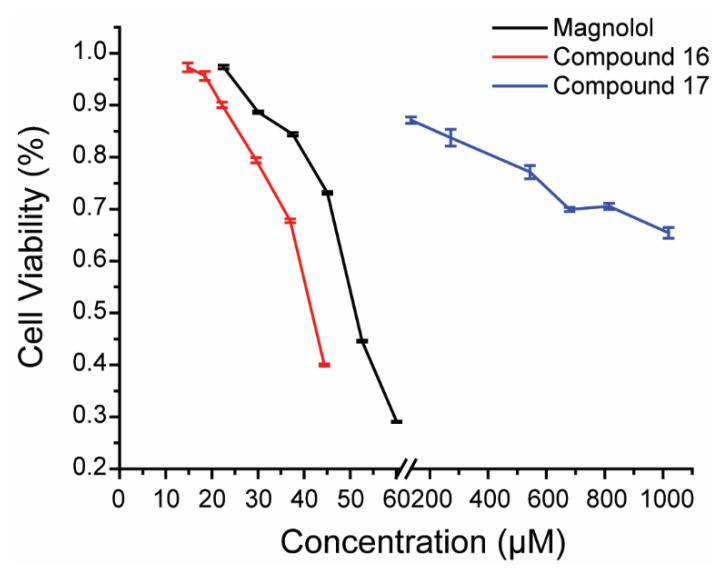
Cytotoxicity of magnolol, compound **16**, and compound **17** toward GCO cells. Data are presented as the mean ± SD.

The cytotoxicity of magnolol, compound **16**, and compound **17** toward GCO cells was measured using a CCK-8 kit, and the results are shown in Figure 3. The 20% cytotoxic concentration and corresponding inhibitory rate are reported in Table 2. According to Table 2, only magnolol and compound **16** showed satisfying anti-MSRV activity at CC_20_, with values of 90.69% and 99.59%, respectively. On the other hand, compound **17** had the lowest inhibitory rate of 43.85% and was considered ineffective. Thus, magnolol and compound **16** were chosen for the subsequent experiments.

### 3.3. Antiviral Activity of Magnolol and Compound ***16*** against MSRV In Vitro

**Figure 4:**
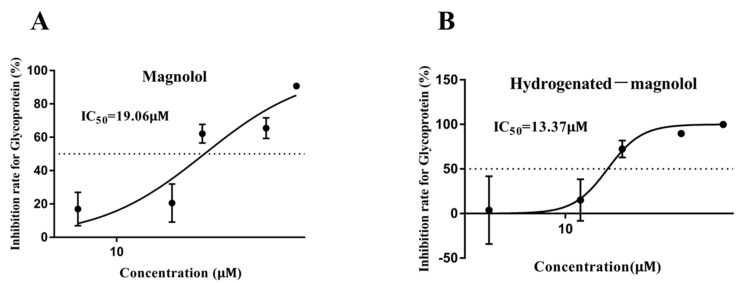
Dose–dependent effect of magnolol (**A**) and hydrogenated magnolol (**B**) against MSRV. The half–maximal inhibitory concentration (IC_50_) is marked. The error bar indicates the SEM.

Different concentrations of magnolol and compound **16** were used to evaluate the dose–effect of these compounds against MSRV on GCO cells by measuring the expression of viral G protein. As shown in Figure 4, both magnolol and hydrogenated magnolol inhibited MSRV replication in a dose-dependent manner. The 48 h IC_50_ of magnolol and hydrogenate magnolol against MSRV was 19.06 μM and 13.37 μM, respectively.

**Figure 5:**
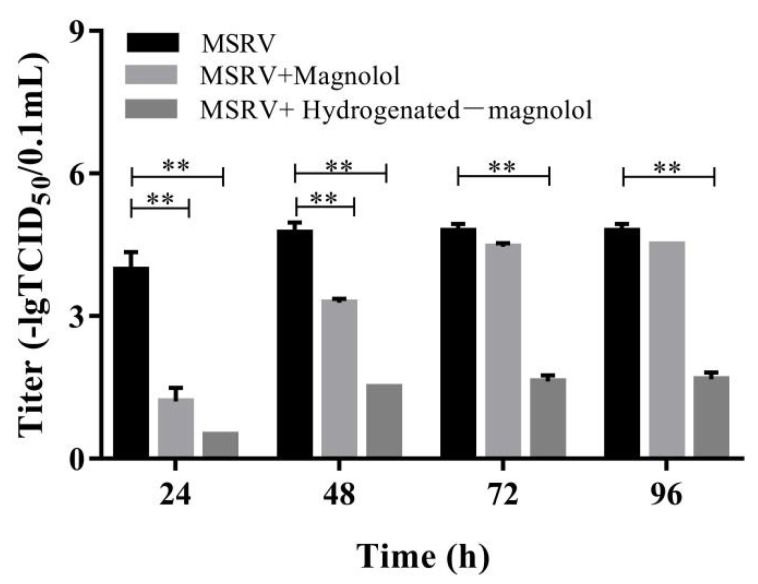
Effects of magnolol and hydrogenated magnolol on MSRV titers. GCO cells infected with MSRV were treated with or without magnolol/hydrogenated magnolol (28.89 μM), and viral titers were calculated using the Karber method at the indicated times. The error bar indicates the SEM. ** *p* < 0.01.

In addition, we further investigated the titers of MSRV after magnolol and hydrogenated magnolol treatment for various times, ranging from 24 h to 96 h. Consistent with the results of qRT-PCR, significant inhibition of MSRV was observed in magnolol- and hydrogenated magnolol-treated GCO cells by measuring the viral titers. As shown in Figure 5, magnolol and hydrogenated magnolol could both decrease the viral titer significantly compared with the control groups. However, from 72 h to 96 h, only hydrogenated magnolol showed viral protection compared with the magnolol and control groups. These results indicated that MSRV replication could be significantly inhibited by magnolol and hydrogenated magnolol treatment, while hydrogenated magnolol had a better protective effect than magnolol. 

### 3.4. Protective Effect of Magnolol and Compound ***16*** on GCO Cells with MSRV Infection

**Figure 6:**
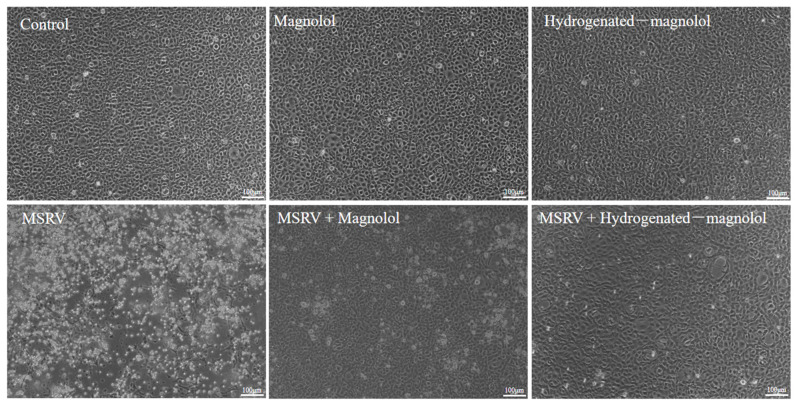
Effects of magnolol and hydrogenated magnolol on CPE induced by MSRV. Hydrogenated magnolol inhibited the CPE phenomenon better than magnolol at the same concentration of 28.89 μM.

Rhabdovirus infection can induce severe cellular and nuclear damage of host cells [18]. Figure 6 shows that MSRV infection could induce significant CPE and cell death in GCO cells. When virus-infected cells were treated with magnolol or hydrogenated magnolol, no obvious CPE phenomenon or nuclear damage was observed. Moreover, hydrogenated magnolol could inhibit MSRV infection better than magnolol. This result demonstrated that hydrogenated magnolol exhibited highly efficient inhibition of MSRV-induced cell death. 

### 3.5. Antiviral Activity of Hydrogenated Magnolol on MSRV Infection Steps in GCO Cells

**Figure 7:**
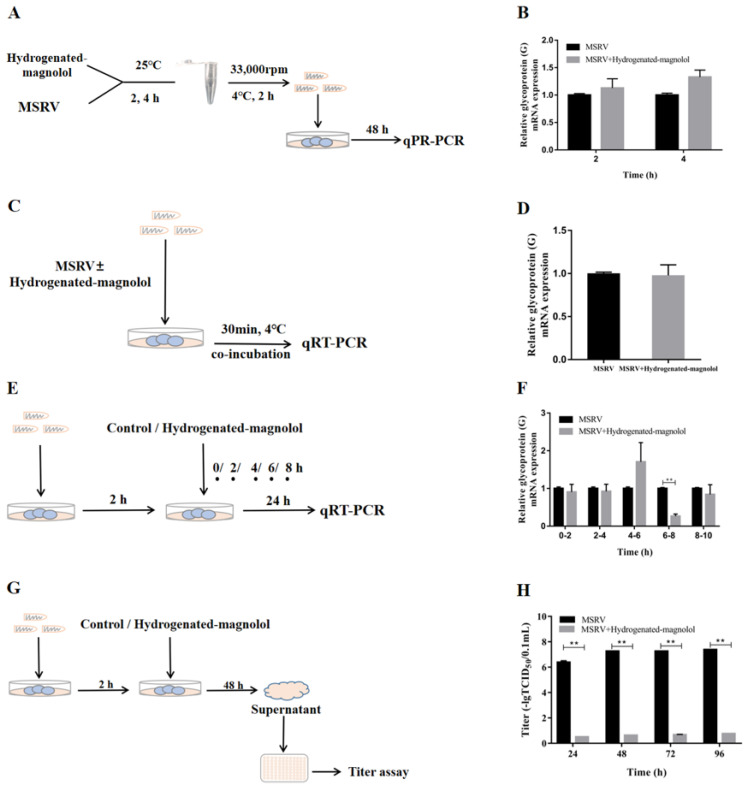
Effect of hydrogenated magnolol on MSRV infection steps in GCO cells. (**A**,**C**,**E**,**G**) illustrate the workflows of the assay design followed in (**B**,**D**,**F**,**H**), respectively. (**B**) The infectivity of MSRV was not affected by hydrogenated magnolol. (**D**) MSRV adhesion was not influenced by hydrogenated magnolol. (**F**) The replication of MSRV in the 6–8 h period was inhibited by hydrogenated magnolol. (**H**) The release of MSRV particles in GCO cells was restrained by hydrogenated magnolol. Relative glycoprotein G gene expression was analyzed by qRT-PCR and calculated on the basis of the 2^−ΔΔCt^ method. The viral titers in the supernatant were calculated using the Karber method at the indicated times. Error bars indicate the SEM. ** *p* < 0.01.

To investigate the anti-MSRV mechanism of magnolol and hydrogenated magnolol, different experimental setups were designed. First, we investigated whether hydrogenated magnolol had a direct impact on MSRV particles. MSRV particles were incubated with 28.89 μM compound **16** for 2 h and 4 h in vitro and recovered by ultracentrifugation before inoculation of cultures (Figure 7A). As shown in Figure 7B, MSRV G gene expression was not significantly changed, which indicated that incubation of the virus with hydrogenated magnolol did not directly impact the viral particles. To further study the mechanism of hydrogenated magnolol against MSRV, we analyzed whether hydrogenated magnolol could interfere with virus adsorption by host cells (Figure 7C). As shown in Figure 7D, hydrogenated magnolol did not affect viral adhesion.

Since hydrogenated magnolol exhibited no obvious influence on MSRV infectivity and binding, a time-of-addition study was performed by exposing MSRV-infected cells to hydrogenated magnolol at different phases of the virus replication cycle (Figure 7E). The results showed that when hydrogenated magnolol was added 6–8 h after viral infection, viral G protein expression levels were significantly decreased by 72.7%. Meanwhile, no inhibition was observed at 0–2 h, 2–4 h, 4–6 h, and 8–10 h (Figure 7F). These results suggested that the time of MSRV replication in the initial cycle was 6–8 h, and the replication of MSRV was suppressed by hydrogenated magnolol. To further study the mechanism of hydrogenated magnolol against MSRV, the effect of hydrogenated magnolol on the release of virus particles was examined (Figure 7G). The viral titer of supernatants from MSRV-infected cells was significantly reduced after treatment with hydrogenated magnolol for 24–96 h (*p* < 0.05, Figure 7H), which indicated that hydrogenated magnolol inhibited the release of the virus.

### 3.6. In Vivo Antiviral Activity of Magnolol and Hydrogenated Magnolol

**Figure 8:**
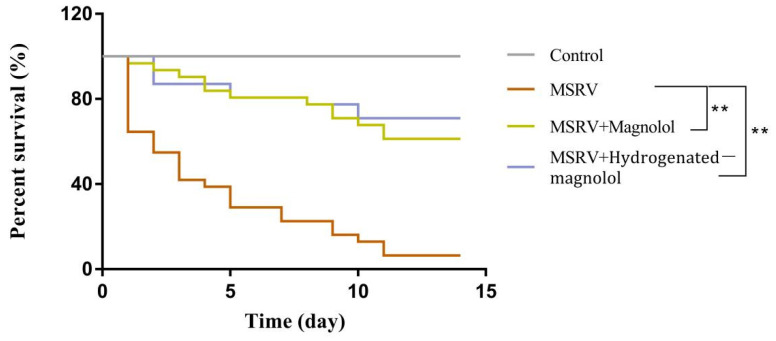
Survival curves of fish intraperitoneally injected with MSRV (1 × 10^6^ TCID_50_) and magnolol/hydrogenated magnolol at a concentration of 20 mg/kg. ** *p* < 0.01.

In this assay, the cumulative mortality of fish was determined in order to evaluate the antiviral effect of magnolol and hydrogenated magnolol against MSRV infection during the 15 day observation period. After intraperitoneal injection with 20 mg/kg magnolol or hydrogenated magnolol, the survival rate of largemouth bass was increased by 44.6% and 62.7% compared to fish only infected with MSRV, respectively (Figure 8). 

**Figure 9:**
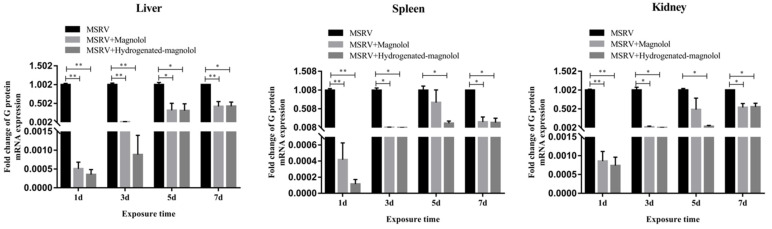
Expression of MSRV G gene in liver, spleen, and kidney after injection with MSRV and magnolol/hydrogenated magnolol (20 mg/kg). Each value represents the mean ± SEM. ** *p* < 0.01, * *p* < 0.05.

Furthermore, the viral load of MSRV in the liver, spleen, and kidney in the magnolol- and hydrogenated magnolol-treated groups was significantly decreased compared with MSRV-infected groups on days 1, 3, 5, and 7 (Figure 9). The above results showed that magnolol and hydrogenated magnolol could effectively inhibit MSRV infection for largemouth bass. 

## 4. Discussion

Largemouth bass, as a famous global economic fish, has been extensively cultured all over the world, due to advantageous traits such as delicious meat, strong disease resistance, and fast growth rate [19]. Since it was first introduced in China in the 1980s, this fish quickly underwent culture in many provinces such as Guangdong, Sichuan, and Zhejiang. To date, the fish has mainly been cultured in an intensive culture system, with the average yield reaching about 37,500 kg/ha. However, with the increase in stocking density and the massive use of artificial feed, outbreaks of viral diseases have acted as major limiting factors for largemouth bass farming [20,21]. MSRV has been confirmed to be one of the most prevalent and virulent pathogens, causing heavy economic losses [4]. MSRV infection was revealed to cause >40% mortality of largemouth bass fingerlings; however, its mechanism of infection has remained largely explored [22]. Moreover, like MSRV, other fish rhabdoviruses, including spring viremia carp virus (SVCV), viral hemorrhagic septicemia virus (VHSV), pike fry rhabdovirus (PFRV), perch rhabdovirus (PRV), ulcerative disease rhabdovirus (UDRV), hirame rhabdovirus (HIRRV), and *Siniperca chuatsi* rhabdovirus (SCRV), can infect marine and freshwater fish, causing great economic losses in the aquaculture industry [23,24,25,26,27]. Currently, no drugs are available to prevent and control MSRV infection [28,29]. The discovery and the development of new antiviral drugs are usually costly and time-consuming processes. Initial steps in novel drug discovery involve the identification of new chemical entities (NCEs). To find NCEs, drug screening from herbs is one of the most important approaches, and the sources of many of the new drugs and active ingredients of medicines are derived from natural products. The starting step for novel plant-based drug discovery should be the identification of the right candidate plants. Substantial research has been conducted to search for useful herbal medicines or to isolate and identify active compounds from these herbs for the prevention and control of viral diseases [30]. For example, Micol et al. found that extracts from olive tree leaf (*Olea europaes*) inhibited the in vitro infectivity of VHSV, a salmonid rhabdovirus [31]. Furthermore, extracts from *Punica granatum* [32], emodin, and barbaloin [33] have been reported to show anti-rhabdovirus activity. Our research group found that arctigenin from *Fructus arctii* showed activity against SVCV [34] and multiple rhabdoviruses (IHNV and SVCV) in aquaculture [7]. However, few studies have focused on drug screening for anti-MSRV infection. Recently, Wang et al. reported drug screening for anti-MSRV from 10 FDA-approved common antiviral drugs, namely, 1-adamantanamine, 1-adamantanamine, 1-adamantanamine, ribavirin, moroxydine hydrochloride, acyclovir, ganciclovir, vidarabine monohydrate, isoprinosine, and *N*2,9-diacetylguanine. The results showed that ribavirin exhibited the best antiviral activity, with a 98.33% inhibition rate at 17.32 μM, and significantly increased cell viability [15]. Searching for drugs to treat emerging viral diseases among FDA-approved antiviral drugs is a quick and useful approach, because the scalable production, antiviral mechanism of action, and side-effects of these drugs are well studied. However, great caution should be paid when considering the use of these drugs for aquaculture. Currently, no drugs have been approved by the FDA for application in fisheries and aquaculture for viral disease treatment [35], since many of these drugs are from drug classes considered “critically important” or “highly important” for human medicine and have been found to be possible drug-resistant strains when used in aquaculture production [36,37].

Most aquaculture production comes from developing countries and depends on a traditional system of medicines used for a variety of diseases [38]. Herbs have been widely used in both veterinary and human medicine. They are natural products that are not only safe for consumers but also widely available throughout Asia. Today, herbs or herbal products also play a significant role in aquaculture. Several hundred plant genera are used medicinally and are vital sources of potent and powerful drugs. Herbs are rich in a wide variety of secondary metabolites of phytochemical constituents such as tannins, alkaloids, flavonoids, and glycosides [39] against different diseases. 

Magnolol is small-molecule polyphenol isolated from the bark of *Magnolia officinalis*, which is widely used in traditional Chinese medicines. Magnolol displays a wide range of biological activities, including antioxidative, antimicrobial, antifungal, and anticancer activity [40]. Our previous studies showed that magnolol could enhance antiviral immune responses against GCRV [11] and protect *Ctenopharyngodon idella* kidney cells from apoptosis induced by GCRV [12]. In addition, Amblard et al. found that magnolol exhibited specific anti-proliferation activity and moderate anti-HIV-1 activity in primary human lymphocytes [41]. Many signaling pathways including NF-κB/MAPK, ROS-mediated apoptosis, mTOR, Nrf2/HO-1, and PI3K/AKT pathways are implicated in the biological functions mediated by magnolol [42]. Both findings demonstrated that magnolol could be used as a lead compound for drug development. However, its content is too low when extracted and purified from the crude extract. Structural optimization and modification of the structure of magnolol is a promising and useful approach to increase its anti-MSRV activity via the synthesis of novel magnolol derivatives. Within this context, a pharmacophore model of magnolol was predicted by Phase module 3.5 software, showing the pharmacophoric features of the phenolic hydroxyl group and propylene group. Thus, in this study, we synthesized two different kinds of magnolol derivatives. One derivative involved replacing the phenolic OH group with a methoxy group (compound **17**), while the other magnolol derivative involved the hydrogenation of the propylene group (compound **16**). The anti-MSRV activity of these derivatives showed that the cytotoxicity of compound **17** against GCO cell was significantly increased compared to its parental natural compound. Hydrogenation of magnolol decreased the viral titer and exhibited enhanced anti-MSRV activity, with an IC_50_ of 13.37 μM. Baschieri et al. also found that the presence of hydroxylated substituents in magnolol derivatives could affect the antioxidant activity of the resulting compounds [43]. Maioli et al. revealed that the presence of a free phenol OH group is a key point in the cytotoxicity observed in HepG2 cells [44]. No cytotoxic effect was observed in our experimental conditions toward GCO cells for hydrogenated magnolol. Studies have indicated that hydroxyl groups in a compound structure affect the interaction between molecules and biological macromolecules, thus affecting their bioactivity [45]. According to the results from the abovementioned research, the phenolic hydroxyl group and aromatic ring are important active sites in magnolol. The increased anti-MSRV activity of hydrogenated magnolol implies that the hydroxyl group will likely figure prominently in synthesizing magnolol derivatives. Any functional disturbance of the hydroxyl group could reduce anti-MSRV activity. 

In conclusion, magnolol derivatives were designed and synthesized on the basis of two different pharmacophore hypotheses. Their anti-MSRV activities were evaluated both in vitro and in vivo. Substituting the phenolic hydroxyl group in magnolol with a methoxy group greatly increased its cytotoxicity toward viral host GCO cells and decreased the anti-MSRV activity to 43.85% at CC_20_. In contrast to modifying the phenolic hydroxyl group, when the propylene group was hydrogenated, this new compound showed enhanced anti-MSRV activity with an IC_50_ of 13.37 μM (vs. magnolol IC_50_ = 19.06 μM). Moreover, hydrogenated magnolol could protect viral host GCO cells by reducing the CPE induced by MSRV infection and significantly reducing the viral G gene expression. Hydrogenated magnolol was found to not directly impact MSRV viral particles or viral adsorption. A single intraperitoneal injection of 20 mg/kg hydrogenated magnolol increased the survival rate of largemouth bass by 62.7% compared to the MSRV-infected groups, significantly reducing viral loads in the liver, spleen, and kidney of fish (*p* < 0.05). Overall, structural optimization of magnolol by hydrogenation of the propylene group increased its anti-MSRV activity both in vitro and in vivo. This finding may provide a valuable reference for the discovery and development of anti-MSRV drugs in aquaculture. 

## Figures and Tables

**Table 1:** Sequences of primers for this study.

Genes		Primer Sequences (from 5′ to 3′)
MSRV glycoprotein (G)	Forward	TGTCAATGTGCGGAGAGGTG
	Reverse	TGTGATACGTAGCTGAGCCG
GCO cells β-actin	Forward	GATGATGAAATTGCCGCACTG
	Reverse	ACCGACCATGACGCCCTGATGT
Largemouth bass β-actin	Forward	CCACCACAGCCGAGAGGGAA
	Reverse	TCATGGTGGATGGGGCCAGG

**Table 2:** The 20% cytotoxic concentration (CC_20_) of three compounds toward GCO cells (±95% confidence interval) and their inhibitory rate of MSRV proliferation at CC_20_.

Compound	Structure	CC_20_ (μM)	Inhibition Rate at CC_20_
Magnolol	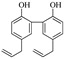	38.86 (30.00–43.89)	90.69%
Hydrogenated magnolol (**16**)	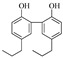	28.89 (26.82–30.74)	99.59%
2,2′-dimethoxy-magnolol (**17**)	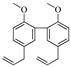	350.90 (201.20–472.09)	43.85%

## Data Availability

Not Applicable.

## Supplementary Materials

The following supporting information can be downloaded at: https://www.mdpi.com/article/10.3390/v14071421/s1, Figure S1. Pharmacophore hypotheses of Magnolol modeled by Phase module 3.5 (Schrödinger software) showed the pharmacophoric features of phenolic hydroxyl group and propylene group.
