# Supplementary material for: Synthesized Magnolol Derivatives Improve Anti-Micropterus salmoides Rhabdovirus (MSRV) Activity In Vivo
Source: Viruses. 2022 Jun 28;14(7):1421. doi: 10.3390/v14071421 (PMC9324556; doi:10.3390/v14071421)
Supplement: Supplementary file 1 [file viruses-14-01421-s001.zip › viruses-1770092-supplementary.pdf]

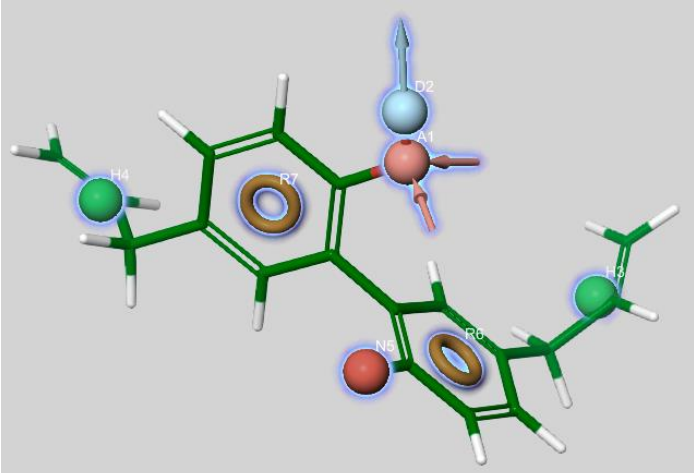

Figure S1. Pharmacophore hypotheses of Magnolol modeled by Phase module 3.5 (Schrödinger software) showed the pharmacophoric features of phenolic hydroxyl group and propylene group. Abbreviations: A, hydrogen bond acceptor; D, hydrogen bond donor; H, hydrophobic group; R, aromatic ring.
